# Supplementary material for: The small GTPase Rab5 inhibits actin polymerization mediated by the Legionella pneumophila effector VipA
Source: Med Microbiol Immunol. 2026 Apr 2;215(1):10. doi: 10.1007/s00430-026-00871-5 (PMC13046614; doi:10.1007/s00430-026-00871-5)
Supplement: Supplementary file 1 — Supplementary file1 (DOCX 279 KB) [file 430_2026_871_MOESM1_ESM.docx]

**Supplementary Material**

**The small GTPase Rab5 inhibits actin polymerization mediated by the *Legionella pneumophila* effector VipA**

Joana Saraiva^1*^, Joana N. Bugalhão^1*^, Teresa Carvalho^1,3^, Zach Hensel^3^, Alvaro Crevenna^3^, L. Jaime Mota^1,2^, Irina S. Franco^1,2^

^1^ UCIBIO – Applied Molecular Biosciences Unit, Department of Life Sciences, NOVA School of Science and Technology, NOVA University Lisbon, Caparica, Portugal

^2^ Associate Laboratory i4HB - Institute for Health and Bioeconomy, NOVA School of Science and Technology, NOVA University Lisbon, Caparica, Portugal

^3^ ITQB NOVA, NOVA University Lisbon, Oeiras, Portugal

^*^ these authors contributed equally to this work

**Corresponding author:** Irina Saraiva Franco, irinafranco@fct.unl.pt

**TABLES**

**Table S1.** Bacterial strains and plasmids used in this work.

| ***E. coli* strains** | **Purpose** | **Reference** | |
| --- | --- | --- | --- |
| **NEB 10b** | Plasmid cloning | New England Biolabs | |
| **BL21(DE3)** | Overexpression and purification of His_6_- and GST-tagged proteins | [41] | |
| **BTH101** | Bacterial Two-Hybrid assays | [42] | |
| **Plasmid** | **Purpose/Construction** | **Reference** | |
|  | ***Transfection of mammalian cells*** |  | |
| **pEF6/Myc-His a** | Vector for C-terminal myc fusions | Invitrogen | |
| **pIF328** | pEF6-*vipA*; encodes VipA_WT_-myc | [31] | |
| **pIF344** | pEF6-*vipA_ΔNH2_*; encodes VipA_ΔNH2_-myc | [31] | |
| **pIF361** | pEF6-*vipA_ΔCOOH_*; encodes VipA_ΔCOOH_-myc | [31] | |
| **pIF368** | pEF6-*vipA_ΔCC_*; encodes VipA_ΔCC_-myc | [31] | |
| **pEGFP-c1** | Vector for N-terminal EGFP fusions | Clontech | |
| **pIF398** | pEGFP-C1-*rab5^W^*^T^; encodes EGFP-Rab5^WT^  (region amplified by PCR from pmRFP-Rab5 with oligos 2072 and 2073, digested with HindIII-BamHI and inserted into pEGFP-c1 HindIII-BamHI) | This work | |
| **pEGFP-C1-*rab5^Q79L^*** | pEGFP-C1-*rab5^CA^*; encodes EGFP-Rab5^CA^ | [43] | |
| **pEGFP-C1-*rab5^S34N^*** | pEGFP-C1-*rab5^DN^*; encodes EGFP-Rab5^DN^ | [43] | |
| **pmRFP-Rab5** | DNA template for *rab5^WT^* PCRs | [44] | |
|  | ***Bacterial Two-Hybrid (BACTH)*** | |  |
| **pUT18C** | Vector for fusions to the C-terminus of fragment T18 | | [42] |
| **pKT25** | Vector for fusions to the C-terminus of fragment T25 | | [42] |
| **pUT18** | Vector for fusions to the N-terminus of fragment T18 | | [42] |
| **pKNT25** | Vector for fusions to the N-terminus of fragment T25 | | [42] |
| **pUT18C-zip** | Positive control for BACTH assays; encodes T18-ZIP | | [42] |
| **pKT25-zip** | Positive control for BACTH assays; encodes T25-ZIP | | [42] |
| **pJA1** | pKNT25-rab5WT; encodes Rab5^WT^-T25 (region amplified by PCR from pIF398 by PCR with oligos 2269 and 2270, digested with PstI-KpnI and inserted into pKNT25 PstI-KpnI) | | This work |
| **pJA2** | pUT18-vipA; encodes VipA-T18 (region amplified by PCR from *L. pneumophila* JR32 with oligos 2266 and 2267, digested with PstI-KpnI and inserted into pUT18 PstI-KpnI) | | This work |
| **pJA3** | pUT18-rab5WT; encodes Rab5^WT^-T18 (region amplified by PCR from pIF398 by PCR with oligos 2269 and 2270, digested with PstI-KpnI and inserted into pUT18 PstI-KpnI) | | This work |
| **pJA4** | pKNT25-vipA; encodes VipA-T25 (region amplified by PCR from *L. pneumophila* JR32 with oligos 2266 and 2267, digested with PstI-KpnI and inserted into pKNT25 PstI-KpnI) | | This work |
| **pJA5** | pKT25-vipA; encodes T25-VipA (region amplified by PCR from *L. pneumophila* JR32 with oligos 2265 and 2267, digested with PstI-KpnI and inserted into pKT25 PstI-KpnI) | | This work |
| **pJA6** | pKT25-*rab5^W^*^T^; encodes T25-Rab5^WT^ (region amplified by PCR from pIF398 by PCR with oligos 2268 and 2270, digested with PstI-KpnI and inserted into pKT25 PstI-KpnI) | | This work |
| **pIF407** | pUT18C-rab5WT; encodes Rab5^WT^-T18 (region amplified by PCR from pIF398 by PCR with oligos 2269 and 2270, digested with PstI-KpnI and inserted into pUT18C PstI-KpnI) | | This work |
| **pIF408** | pUT18C-*vipA_WT_*; encodes T18-VipA_WT_ (region amplified by PCR from *L. pneumophila* JR32 with oligos 2266 and 2267, digested with PstI-KpnI and inserted into pUT18C PstI-KpnI) | | This work |
| **pIF409** | pKT25-*rab5^CA^*; encodes T25-Rab5^CA^ (region amplified by PCR from pEGFP-C1-*rab5^Q79L^* by PCR with oligos 2268 and 2270, digested with PstI-KpnI and inserted into pKT25 PstI-KpnI) | | This work |
| **pIF410** | pKT25-*rab5^DN^*; encodes T25-Rab5^DN^ (region amplified by PCR from pEGFP-C1-*rab5^S34N^* by PCR with oligos 2268 and 2270, digested with PstI-KpnI and inserted into pKT25 PstI-KpnI) | | This work |
| **pIF413** | pUT18C-*vipA_ΔCOOH_*; encodes T18-VipA_ΔCOOH_ (region amplified by PCR from pIF408 with oligos 2266 and 2329, digested with PstI-KpnI and inserted into pUT18C PstI-KpnI) | | This work |
|  | ***Protein Overexpression and Purification*** |  | |
| **pGEX-4T-2** | Vector for N-terminal GST fusions | GE Healthcare | |
| **pIF415** | pGEX-*rab5^WT^*; encodes GST-Rab5^WT^ (region amplified from pIF398 by PCR with oligos 2331 and 2332, digested with EcoRI-XhoI and inserted into pGEX-4T-2 EcoRI-XhoI) | This work | |
| **pIF416** | pGEX-*rab5^CA^;* encodes GST-Rab5^CA^ (region amplified from pEGFP-C1-*rab5^Q79L^* by PCR with oligos 2331 and 2332, digested with EcoRI-XhoI and inserted into pGEX-4T-2 EcoRI-XhoI) | This work | |
| **pIF417** | pGEX-*rab5^DN^*; encodes GST-Rab5^DN^ (region amplified from pEGFP-C1-*rab5^S34N^* by PCR with oligos 2331 and 2332, digested with EcoRI-XhoI and inserted into pGEX-4T-2 EcoRI-XhoI) | This work | |
| **pET15b-*vipA*** | pET15b-His_6_-VipA_WT_ | [26] | |
| **pIF358** | His_6_-VipA_∆NH2_ (region amplified by PCR with oligos 1592 and 1591, digested with NdeI-BamHI and inserted into pET28b) | [31] | |
| **pIF373** | His_6_-VipA_∆COOH_ (region amplified by PCR with oligos 1590 and 1299, digested with NdeI-BamHI and inserted into pET28b) | [31] | |
| **pIF374** | His_6_-VipA_∆CC_ (region amplified by PCR with oligos 1590 and 1591, digested with NdeI-BamHI and inserted into pET28b) | [31] | |
| **pJB9** | His_6_-VipA_COOH_ (region amplified by PCR with oligos 1775 and 1591, digested with NdeI-BamHI and inserted into pET28b) | [31] | |

**Table S2.** Oligonucleotides used in this work

| **Oligonucleotide** | **Sequence (5’→3’) *^a^*** |
| --- | --- |
| 2265 | AAAACTGCAGGGATGCCTATCAGTAATGCCTTTC |
| 2266 | AAAACTGCAGGATGCCTATCAGTAATGCCTTTC |
| 2267 | AAAAGGTACCCGGAGATTTTTTTTTTCGACGGG |
| 2268 | AAAACTGCAGGGATGGCTAGTCGAGGCGCAAC |
| 2269 | AAAACTGCAGGATGGCTAGTCGAGGCGCAAC |
| 2270 | AAAAGGTACCCGGTTACTACAACACTGATTCC |
| 2328 | AAAACTGCAGGTTTATTTTAGCTCAAAAGGC |
| 2329 | AAAAGGTACCCTATGTGATTTCGCTTAGAGTTTG |
| 2330 | AAAAGGTACCCTACGCTTGTTGCGTCATGACCAG |
| 2072 | AAAAAAGCTTCTATGGCTAGTCGAGGCGCAACAAG |
| 2073 | TCCGGTGGATCCTTAGTTACTACAACACTG |
| 2331 | AAAAGAATTCCCATGGCTAGTCGAGGCGCAACAAG |
| 2332 | AAAACTCGAGTTAGTTACTACAACACTGATTCC |

*^a^* Restriction sites are underlined.
