# Supplementary material for: The small GTPase Rab5 inhibits actin polymerization mediated by the Legionella pneumophila effector VipA
Source: Med Microbiol Immunol. 2026 Apr 2;215(1):10. doi: 10.1007/s00430-026-00871-5 (PMC13046614; doi:10.1007/s00430-026-00871-5)
Supplement: Supplementary file 2 — Supplementary file2 [file 430_2026_871_MOESM2_ESM.pdf]

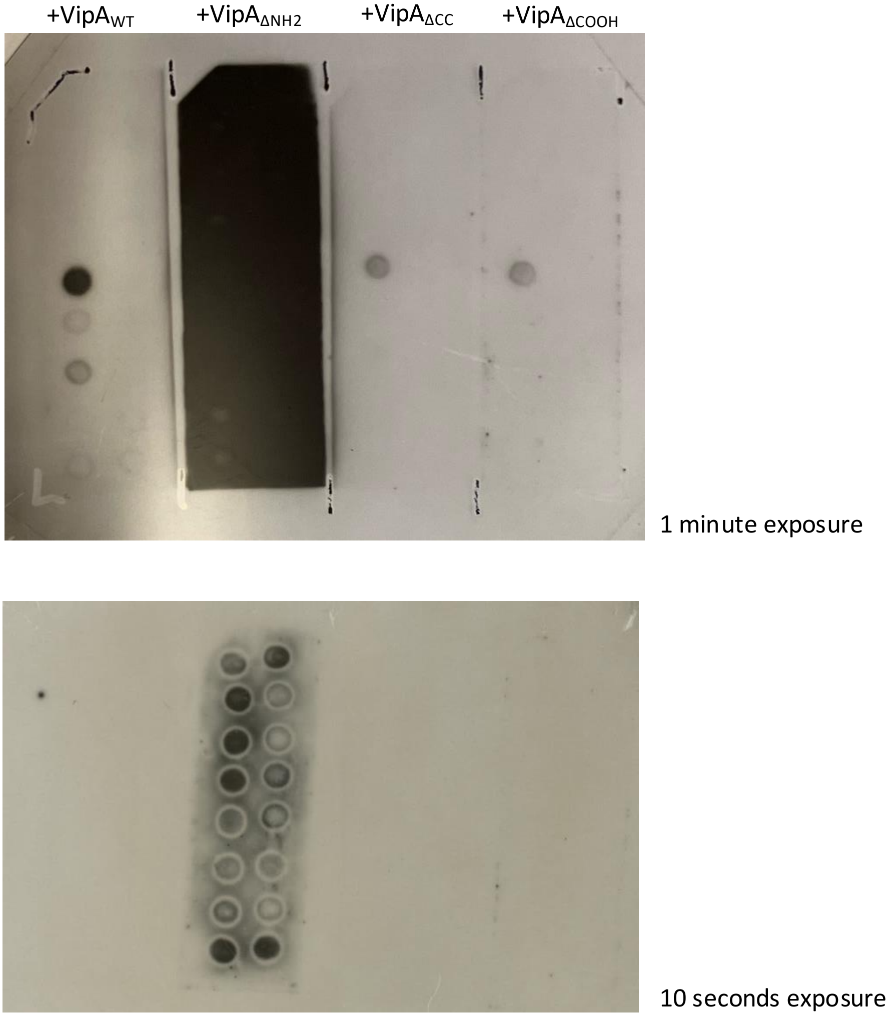

Fig. S1. Specific binding of VipA to PI3P requires the NH_2 region. Membranes containing the indicated immobilized lipids were incubated with purified recombinant VipA$_{WT}$, VipAΔ$_{NH2}$, VipA$_{\Delta CC}$ and VipA$_{\Delta COOH}$ and probed with polyclonal anti-His antibodies (mouse) and secondary anti-mouse-HRP (goat) antibodies (see Figure 1 for details).

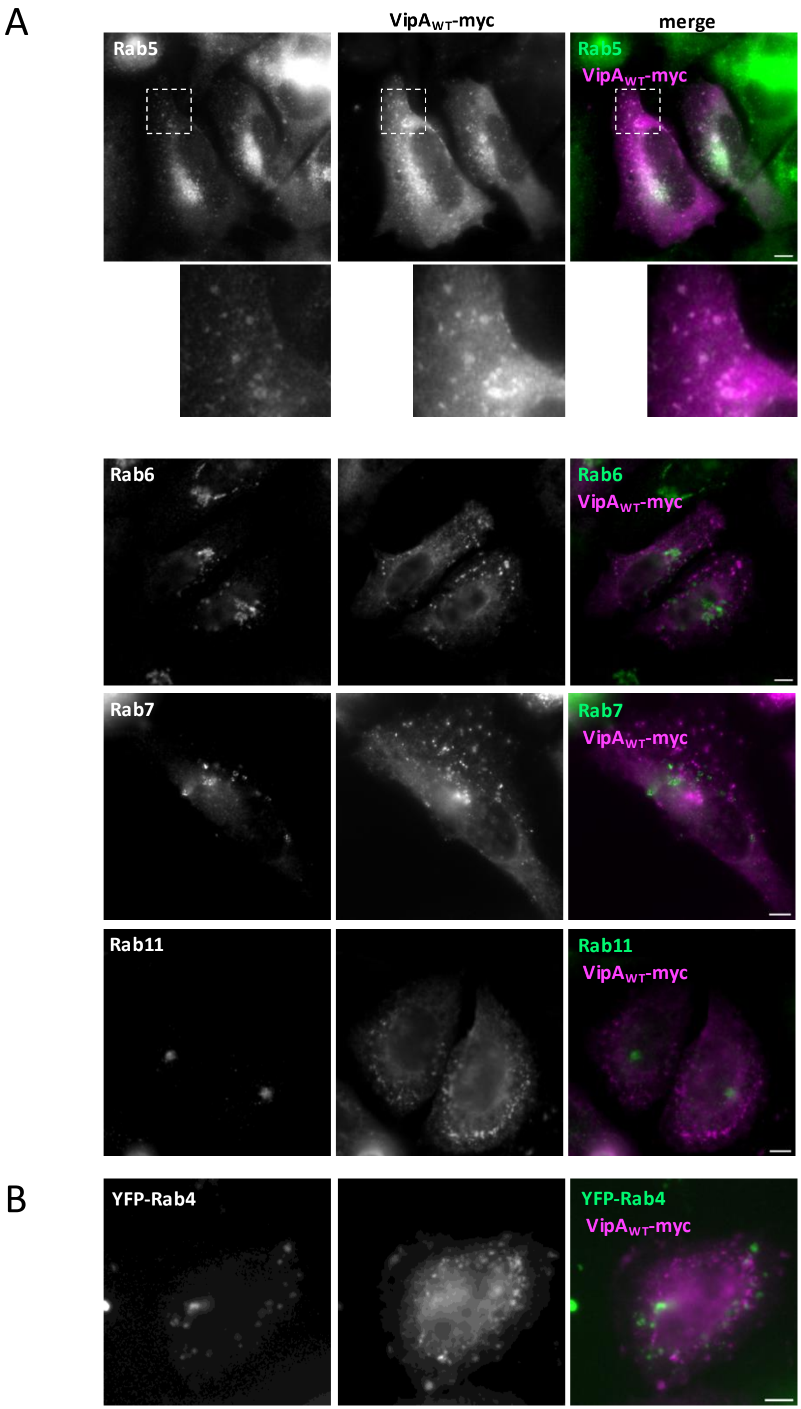

Fig. S2. VipA colocalizes with endosomal Rab GTPase Rab5 in CHO cells. Cells were transfected with a plasmid encoding full-length VipA-myc (A) or co-transfected additionally with plasmid encoding YFP-Rab4 (B), fixed with 4% PFA and permeabilized with 0.1% Saponin, or with methanol in the case of Rab7 labeling. VipA (in magenta) was labeled with anti-myc antibodies (mouse) and secondary anti-mouse-Alexa Fluor 350, and endogenous Rab GTPases were labeled with rabbit anti-Rab5, anti-Rab6, anti-Rab7 or anti-Rab11 and secondary anti-rabbit-Alexa Fluor488. Scale bar, 5 µm.
